# Near-infrared spectroscopy (NIRS) as a new tool for neuroeconomic research

**DOI:** 10.3389/fnhum.2014.00549

**Published:** 2014-08-07

**Authors:** Isabella M. Kopton, Peter Kenning

**Affiliations:** ^1^Department of Corporate Management and Economics, Zeppelin UniversitätFriedrichshafen, Germany; ^2^Faculty of Business Administration and Economics, Heinrich-Heine-UniversitätDüsseldorf, Germany

**Keywords:** mobile fNIRS, prefrontal cortex, real-world setting, neuroeconomics, decision making

## Abstract

Over the last decade, the application of neuroscience to economic research has gained in importance and the number of neuroeconomic studies has grown extensively. The most common method for these investigations is fMRI. However, fMRI has limitations (particularly concerning situational factors) that should be countered with other methods. This review elaborates on the use of functional Near-Infrared Spectroscopy (fNIRS) as a new and promising tool for investigating economic decision making both in field experiments and outside the laboratory. We describe results of studies investigating the reliability of prototype NIRS studies, as well as detailing experiments using conventional and stationary fNIRS devices to analyze this potential. This review article shows that further research using mobile fNIRS for studies on economic decision making outside the laboratory could be a fruitful avenue helping to develop the potential of a new method for field experiments outside the laboratory.

## Introduction

Over the last decade, the investigation of economic research questions by use of well-established neurological and neurophysiological methods such as fMRI, EEG, electrodermal activity (EDA) or eye-tracking has led to the new interdisciplinary research field called “neuroeconomics” (e.g., McClure et al., 2004; Bechara et al., 2005; Camerer et al., 2005; Kenning and Plassmann, 2005; Singer and Fehr, 2005; Brosch and Sander, 2013). In this field, the underlying neurophysiological processes of economic decision making have been increasingly elaborated with diverse research foci.

Different research studies focus on the neural correlates of social dimensions in economic markets (e.g., Fehr et al., 2005; Ruff et al., 2013), explore behavioral game theories through a new neurophysiological perspective (e.g., Sanfey et al., 2003; Bhatt and Camerer, 2005) and investigate brain activities related to investors' financial decision-making behavior (e.g., McClure et al., 2004; Kuhnen and Knutson, 2005). Other research focuses on consumers' decision-making processes and their corresponding brain activities (e.g., Yoon et al., 2006; Knutson et al., 2007; Hedgcock and Rao, 2009). Moreover, management researchers in the area of information system research have begun to use these neurophysiological methods and prior findings to investigate information system constructs, as well as users' decision making in the online world (e.g., Dimoka, 2011; Kopton et al., 2013; Riedl et al., 2014). Recently, management research focusing on organizational behavior has also begun to develop a new interdisciplinary perspective by transferring prior neurological findings to extend organizational theories (e.g., Boyatzis et al., 2014).

This body of research has generally resulted in significant developments by adding a new theoretical perspective to economic research. However, the majority of these well-known neuroeconomic studies, all of which investigate decision making from different economic perspectives (Glimcher et al., 2013), were commonly investigated through fMRI-based research (Braeutigam, 2012).

Nevertheless, fMRI measurements have limitations with regard to how their real-world applicability corresponds to restricted external validity, so that many researchers question whether economic decision making can truly be measured and generalized in such a restricted situation (Shimokawa et al., 2009; Ariely and Berns, 2010; Ayaz et al., 2013). Only limited conditions can be tested in the fMRI-scanner, and only specific types of stimuli can be shown while the participant is lying in the scanner. For this reason, neuroeconomic studies, to date, have primarily focused on “fictive” tasks and not “real-world” situations (Ariely and Berns, 2010). Because of the technical limitations, the influencing stimuli in these studies were often reduced in complexity, suggesting that other measurements are necessary for future field experiments in neuroeconomics.

Against this background, mobile functional near-infrared spectroscopy (fNIRS) measurement seems to have strong potential for applicability in field studies. fNIRS can be defined as a non-invasive optical brain imaging technique that investigates cerebral blood flow (CBF) as well as the hemodynamic response in a local brain area during neural activity (Jackson and Kennedy, 2013). In different prior studies it has been demonstrated that, comparable to functional magnetic resonance imaging (fMRI), the fNIRS method is a reliable and valid measurement for cortical activations (see Ernst et al., 2013).

In response to the limited research, this paper aims to integrate various disciplinary fNIRS studies in economic decision making research which, up to now, have been examined mainly in isolation from each other. Moreover, we provide insight into the potential of fNIRS for measuring brain activations during different real-world situations of economic decision making.

## Recent challenges for neuroeconomics

At present, researchers in (neuro-)economics are challenged by many new economic trends. Particularly, these trends increase the difficulty of using traditional neurophysiological methods such as fMRI to investigate research questions, for which measuring situational factors in the “real world” and outside the laboratory are highly important.

Three current examples of trends regarding consumers' economic decision making effectively illuminate new challenges of (neuro-)economic research:

1. Integrating consumers into the innovation process is becoming increasingly essential (e.g., von Hippel and Katz, 2002; Jeppesen and Molin, 2003; Gassmann et al., 2010). In general, innovative products, ideas or concepts that are considered “radical” or that offer “incremental” developments show high failure rates in economic markets (Hauser et al., 2006), primarily because they often present high risks and costs resulting from uncertainty of consumer or user acceptance (Davis, 1989; Stevens and Burley, 2003; Joshi and Sharma, 2004). Therefore, economic researchers in the area of innovative product development are highly motivated to further investigate determinants of consumer acceptance, perception and preference (e.g., Herrmann et al., 2007; Ariely and Berns, 2010) during integration processes. In classical economic studies, this is often implemented with experiments that use real products and prototypes. The investigation of prototypes and their potential for consumer acceptance is often accomplished through market studies that use real products and prototypes. In general, such investigations raise questions regarding high external validity. These prevailing complexities related to the new trend of integrating real-world consumers into the product innovation process in order to increase certainty of consumers' product acceptance show the necessity for new mobile measurements that can be used outside the laboratory.
2. Another new economic trend is the consumer tendency toward sharing and joint consumption (Bardhi and Eckhardt, 2012). Noting that these issues were a major topic at the 2013 Association for Consumer Research Conference, general indicators suggest that the phenomenon will continue to gain importance. Today, “Generation Y” has fully accepted the innovative trend of collaborative consumption and “non-possession” (Belk, 2010). In contrast to the conventions of earlier generations of consumers who placed value on owning as a sign of prestige, young consumers share such things as clothes, apartments, bicycles and cars, both with known or previously unknown persons (Bardhi and Eckhardt, 2012). Many new business models have emerged in response to this development, and are primarily framed around the idea of allocating market overcapacities (Botsman and Rogers, 2010). This socially based consumer movement will be increasingly relevant for future-oriented economic studies, and thereby will require extension of neuroeconomic theories on consumers' decision making. In order to implement studies concerning the new trend of collaborative consumption, the consumers' interaction in the real world will be an interesting and valuable new perspective. This consequentially arising complexity demands new mobile neuroimaging techniques that can be used for investigating consumers' interactions outside the laboratory.
3. Investigating the operation of companies in new markets is an important area for the expansion of economic studies, and will also require new research methods. For example, an fMRI study about consumer preferences and decision making in new markets presents strong challenges, including the need for cooperation or collaboration with institutes that have easy access to fMRI scanners and clinics. Because of these challenges (particularly in new markets with inadequate infrastructure), new mobile neuroimaging techniques gain in importance and have strong potential, especially in the field of “cultural neuroscience” (Seligman and Kirmayer, 2008).

These three examples of new trends in economic research show the relevance for new methods in (neuro-)economic research outside the laboratory. Overall, neuroeconomic research is challenged by a number of changes that demand new methodological development toward flexible and mobile technologies such as fNIRS.

## Methodological background of fNIRS measurement

An elaboration of the potential of fNIRS methods should begin by detailing the fNIRS methods that are applied for recording data and for analysis. This background both allows useful assessment of the potential of this new method, and brings to light its challenges. Jöbsis (1977) was first to explain how the optical measurement for cerebral hemodynamic response known as NIRS is performed by the irradiation of near-infrared light into participants' head and its scattering position (Villringer et al., 1993; Funane et al., 2013).

Near-infrared light, with a wavelength spectrum of circa 650–950 nm, passes through biological tissue without difficulty, and can non-invasively illuminate several centimeters of the tissue (Lloyd-Fox et al., 2010; Jackson and Kennedy, 2013; Scholkmann et al., 2013). Because of this characteristic transparency, the spectrum of near-infrared light is often called an “optical window” (Jöbsis-vanderVliet, 1999). In general, it can be approximated that oxy-(O2Hb) and deoxy-hemobglobin (HHb) are the main absorbers, so that the changes in oxy- and deoxy-hemoglobin can be assessed, allowing for the indirect quantification of neural activity (Jackson and Kennedy, 2013). Various existing studies calculate the optimal wavelength as well as the optimal number of different wavelengths for perfect illumination based on a mathematical optimization problem (Yamashita et al., 2001; Sato et al., 2004; Correia et al., 2010; Schelkanova and Toronov, 2012; Scholkmann et al., 2013). Based on these physical and mathematical calculations, several techniques have been developed to measure the hemodynamic response. The majority of these studies implement the continuous-wave method (Lloyd-Fox et al., 2010). For oxy-(O2 Hb) and deoxy-hemobglobin (HHb) chromophores, dissimilar near-infrared light absorption properties can be anticipated, so that by using the absorption variation difference resulting from different chemical structure changes in blood oxygenation of the illuminated skin, the skull and some centimeters of brain tissue can be measured (Jöbsis, 1977; Lloyd-Fox et al., 2010).

The near-infrared light sources, which are laser-emitting diodes, are placed directly onto a participant's scalp and are sent—in a “banana-shaped” form (Okada and Delpy, 2003)—to the detectors, called optodes. The depth and exactness of measurement depends on the distance between the source and the detector. In different mathematical models, the correlation of the inter-optode distance and the depth of light penetration are assumed to be proportional (Nossal et al., 1988; Ehlis et al., 2005). However, the larger the distance, the more the light is scattered, so that the detector should not be placed more than 3 cm away from the source (Lloyd-Fox et al., 2010; Jackson and Kennedy, 2013).

**Figure 1:**
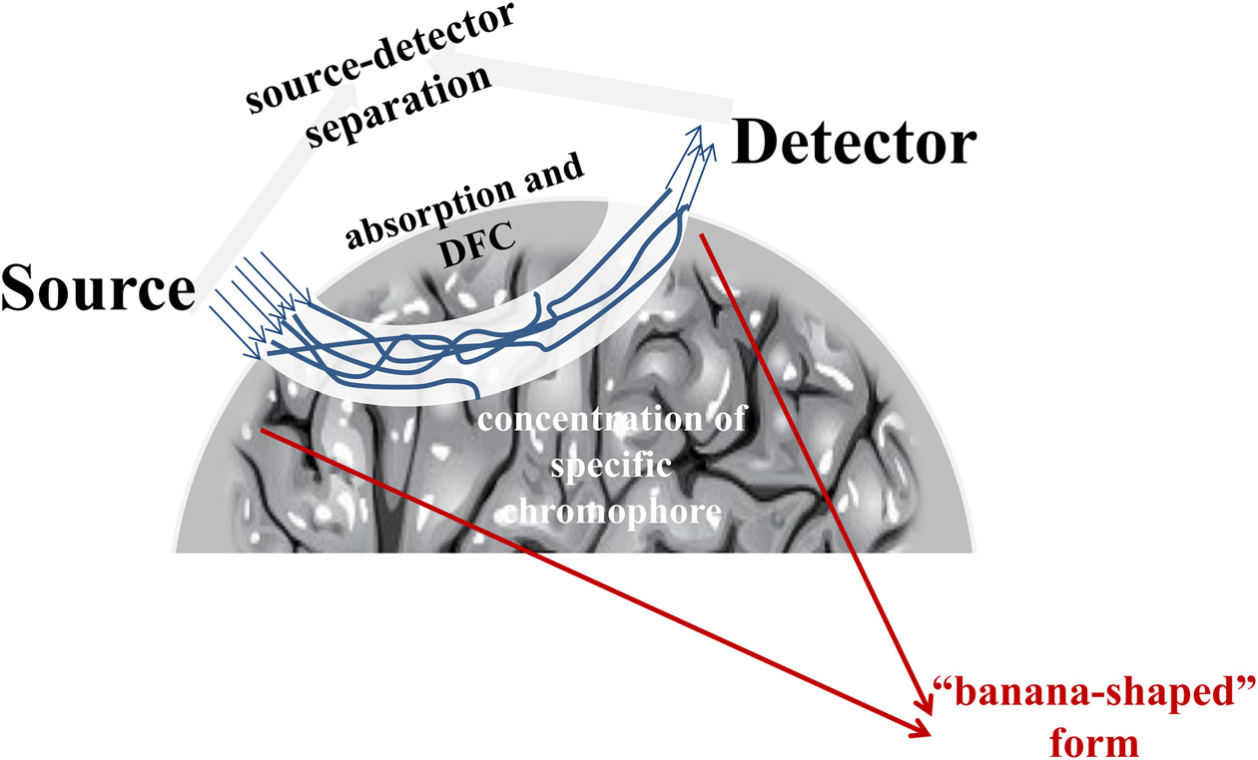
**Change in light attenuation**.

For the conversion of the raw near-infrared light absorption and attenuation data into oxy- and deoxy-hemoglobin concentration, the most commonly used algorithm is the modified Beer–Lambert law (Kocsis et al., 2006; Scholkmann et al., 2013). In contrast to the original Beer–Lambert law, which generally allows the quantification of concentration only for non-scattering media (Scholkmann et al., 2013), the modified Beer–Lambert law considers a constant optical scattering of the light and relates the change in chromophore concentration to the change in light attenuation (see also Figure 1):

ΔA=α∗Δc∗L∗DFC 

(Lloyd-Fox et al., 2010)

with A, light attenuation; α, absorption coefficient; c, concentration of specific chromophore; L, source-detector separation; DFC, differential path length factor, which may vary according to specific wavelength, gender, age and difference in tissue type (Duncan et al., 1995, 1996).

To measure changes in oxy- and deoxy-hemoglobin, the brain needs to be illuminated with two different wavelengths, which in turn need to be integrated into two simultaneous equations in order to measure the blood oxygenation differences in the tissue (Lloyd-Fox et al., 2010).

In addition to the implementation of the modified Beer–Lambert law, fNIRS data analysis requires further preprocessing methods such as motion artifact correction, low- and high-pass filtering (for eliminating breathing, heartbeat and drift; see also Piper et al., 2014) and single-channel signal-to-noise analyses. Similar to fMRI studies, fNIRS data needs to be Bonferroni-corrected for multiple comparisons between channels. This implies that the *p*-values of multiple comparisons are adapted by the number of correlations performed (see also Ernst et al., 2013).

## Literature review of NIRS studies

**Figure 2:**
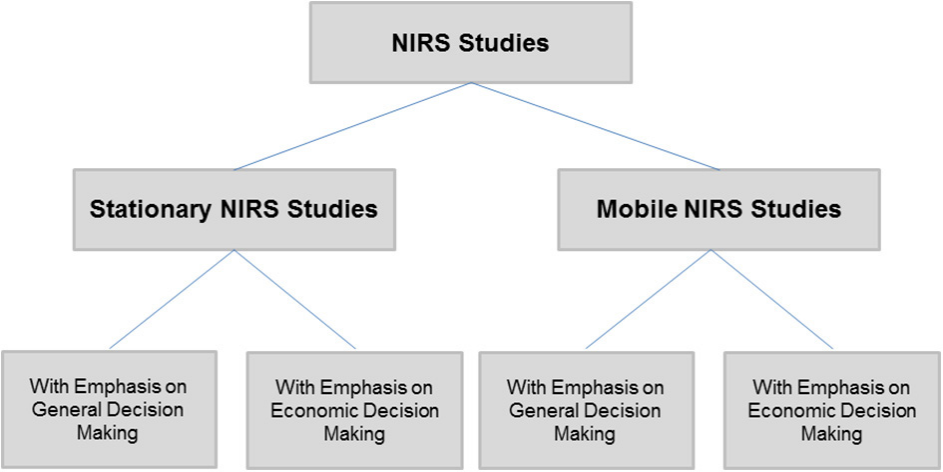
**Literature review: stationary vs. mobile NIRS studies with emphasis on general vs. economic decision making**.

A number of NIRS studies can be found in the literature, some using stationary NIRS machines and others using mobile, wireless and innovative NIRS prototypes. A general NIRS study classification separating “stationary” and “mobile” NIRS has been developed, designating specific subcategories of studies with emphasis on “economic decision making” and on “general decision making” (see Figure 2).

Based on these (Figure 2) classifications, the following section presents various NIRS studies.

### Stationary NIRS studies

#### Stationary NIRS studies with emphasis on general decision making

One group of stationary NIRS studies includes those without a concrete economic research frame, but that explore phenomena that have interest and strong relevance for economic research questions (see Table 1). The following decision making studies, with varied foci, are transferable to economic research questions.

1. Studies transferable to marketing research/transferable to design studies in information system research: Several NIRS studies explore the effects of different visual and auditory stimuli (Köchel et al., 2011; Plichta et al., 2011) on brain activations during participants' decision making. These NIRS studies could effectively complement the neuroeconomic fMRI studies that investigate these various aspects of influencing stimuli, such as the “First-Choice Brand Effect” (Deppe et al., 2005) or the effect of culturally familiar brands on preference (Schäfer et al., 2006). Moreover, these NIRS studies can be transferred to prior fMRI studies that investigate optimal user interface in social networks (Kopton et al., 2013).
2. Studies transferable to leadership research: fNIRS studies dealing with social functioning theory and emotion discrimination effects (Pu et al., 2012, 2013; Schneider et al., 2013) can give new impetus in the area of leadership research and organizational behavior. Few published research studies integrate neurophysiological methods into leadership and organizational research, and the few that do exist are not well known. This is astonishing, because the description, the explanation and configuration of human behavior in organizational systems is both central to and a main aspect of leadership and organizational research (Kenning and Kopton, 2013). Consequently, because interpersonal relationship systems play a major role in the organizational behavior of employees and managers, and in leadership behavior, fNIRS studies (with the potential of implementing field experiments with single trials during real human interpersonal interactions) promise to be highly relevant for this relatively new research area.
3. Studies transferable to interpersonal behavior studies in information systems research: Schneider et al. (2013) designed an experiment using avatar images to investigate participants' brain activations during the judgment of different emotional faces. Riedl et al. (2014) used fMRI machinery to investigate online users' interaction with avatars, as well as with real human beings, in the online world. Though these studies suggest that fNIRS studies can also easily be transferred to information system research, the implementation of NIRS as a tool for real-world settings is not as relevant for “computer interaction” studies. Accordingly, scientists should always deliberate the advantages and disadvantages of using fNIRS devices and fMRI machinery.
4. Studies transferable to decision making studies from strategic management and consumer research perspectives: NIRS studies about decision making under time pressure (Tsujii and Watanabe, 2010) and about decision-making processes investigating approach-avoidance theories (Ernst et al., 2013) are highly relevant for strategic managerial decision making, as well as for consumer decision making (e.g., buying decisions under time pressure). Management studies from various areas such as innovation management research (e.g., Dayan and Elbanna, 2011) and strategic management research (e.g., Sandler-Smith and Shely, 2004) focus on managers' intuitive decisions and “gut feelings” under time pressure. Additionally, in the areas of marketing and consumer research, many studies address questions related to consumer decision making under time pressure (e.g., Reimann et al., 2012; Krishnan et al., 2013). The Ernst et al. (2013) NIRS study on approach-avoidance theory is another area of application.
5. Studies transferable to consumer research studies in the area of compulsive buying behavior/consumer protection research: A number of recent fNIRS studies about ADHD syndrome and other pathological behaviors (Moser et al., 2009; Gehricke et al., 2013) can be readily transferred to consumer research studies in compulsive buying (e.g., Otero-Lopez and Pol, 2013), as well as to consumer protection studies that currently have increasing importance (Lee and Mysyk, 2004; Kenning and Linzmajer, 2011; Kenning and Reisch, 2013).

**Table 1:** **Stationary NIRS studies with emphasis on general decision making**.

**Transfer to marketing research/Transfer to design studies in information systems research**	**Transfer to leadership research/Transfer to interpersonal behavior studies in information systems research**	**Transfer to decision making studies from strategic management and consumer research perspectives**	**Transfer to consumer research studies in the areas of compulsive buying behavior/consumer protection research**
Köchel et al., 2011	Pu et al., 2012, 2013	Tsujii and Watanabe, 2010	Moser et al., 2009
Content: Perception of pictures and imagery Transferable to: Transferable to product and promotion policy; e.g., influence of visual stimuli on advertising and (innovative) products (e.g., Schäfer et al., 2006) as well as to online design studies in Neuro-IS research (Kopton et al., 2013)	Content: Relationship between prefrontal function during a cognitive task and social functioning (motivation factor scores of SASS) Transferable to: Transferable to leadership and personal management research (e.g., investigating working behavior for practical implications; see Kenning and Kopton, 2013)	Content: Time pressure effect and the activity in the inferior frontal cortex (IFC) Transferable to: Managers' strategic decision making under time pressure, as well as consumer decision making under time pressure	Content: Results show that ADHD can be characterized by impairment of the dorsolateral prefrontal cortex Transferable to: Investigating studies of consumers' pathological decision making, such as research about compulsive buying (e.g., Manolis and Roberts, 2008)
Plichta et al., 2011	Schneider et al., 2013	Ernst et al., 2013	Gehricke et al., 2013
Content: Auditory cortex activation is modulated by emotion Transferable to: Transferable to promotion policy and POS-marketing (influence of, for example, music or voice on consumers' decision making; subliminal marketing)	Content: Emotion discrimination task with faceless avatars expressing different patterns (fearful, happy, sad, neutral, angry) and participants' judgment Transferable to: Transferable to managers' interpersonal exchange processes; human resource management with relation to the motivation of employees; interpersonal behavior in the online world (information systems research; e.g., Riedl et al., 2014)	Content: Cortical processes during automatic and regulated approach-avoidance reactions Transferable to: Findings for approach-avoidance theories are transferable to real-world economic decision making of consumers and managers; from a consumer perspective, testing of First Choice Brand Effect (Deppe et al., 2005)	Content: Investigation of how cigarette smoking affects prefrontal brain hemodynamics in smokers with and without ADHD Transferable to: Transferable to prior studies about consumers' pathological decision making (e.g., Otero-Lopez and Pol, 2013)

#### Stationary NIRS studies with emphasis on economic decision making

In the last 5 years a number of new decision making studies investigating concrete and relevant economic research questions were applied to economic decision-making research questions:

1. Investors' risky decision making: The experiments developed by Shimokawa et al. (2009, 2012) investigate investors' decision making processes. The first study (Shimokawa et al., 2009) examines the medial prefrontal cortex (MPFC) and the orbital cortex (OFC) related to risk and reward prediction during decision making, using an fNIRStation from Shimadzu Corporation. In this study, 15 participants fictively received 1 million yen as total assets and were instructed to use a computer to decide a ratio of stock investment. Participants were allowed to change their ratio occasionally, in response to stock prices, which were updated every 750 ms (experimental events). The results of this study show that the OFC is sensitive to responses to price changes (loss prediction), whereas MPFC changes accompany reward predictions. The second study (Shimokawa et al., 2012) confirms these findings regarding expected rewards, and future risks were further developed by investigating the extent to which information about brain activity can advance investment performance during investors' decision making.
2. Consumers' decision making and preferences: In their NIRS experiment, Luu and Chau (2009) address the phenomenon of subjective product preference. Nine adults participated in a computer experiment with a subjective preference task, based on 60 trials (in total) per participant. During these trials the participants were asked to look at two different drinks and to mentally evaluate their preference. The method applied the well-established shopping task of Knutson et al. (2007). For the measurement, a multichannel frequency domain NIRS device with 16 sources and three detectors was used. The results showed that subjective preference could be measured in the MPFC with 80 percent accuracy. Accordingly, this study shows high relevance for the research area of consumer decision making processes, providing new and useful impetus for field experiments on consumer decision making.

### Mobile NIRS studies

#### Mobile NIRS studies with emphasis on general decision making

One of the first mobile near-infrared spectroscopy systems was developed by Bozkurt et al. (2005), for the purpose of continuous monitoring of brain functions for newborns vulnerable to brain injuries (see Table 2). In this study the researchers present the low-cost, battery-operated continuous shot-limited SNR of 67 dB (with dual wavelength) that they had developed for newborns in neonatal intensive care units (NICUs). The phantom study tested the validity and reliability of the NIRS system, and demonstrated the potential of this technology as a clinical tool for measuring the metabolism of newborns in NICUs. Even though this first study had a clinical setting, the development was a first step toward application of a successful mobile tool.

**Table 2:** **Studies with mobile NIRS devices**.

**MOBILE NIRS STUDIES**						
**Authors**	**Area of interest**	**NIRS set-up**	**External validity**	**N**	**Method**	**Results**
Bozkurt et al., 2005	New-born brain metabolism	Validity of the system	Relatively high external validity	1 (new-born) and prior phantom tests	NIRS prototype of a low-cost, battery-operated, dual wavelength, continuous wave	Shot-limited SNR of 67 dB for 10 Hz temporal resolution was achieved. Reliable clinical tool employed at bedside
Muehlemann et al., 2008	Tissue oxygenation and cortical hemodynamic response to sensory stimuli	Wireless NIRI device tested in a solid silicone phantom and an *in-vivo* experiment (4 sources, 4 detectors)	Very low external validity	1 phantom test and 1 male (*in-vivo* experiment)	*In-vivo* experiment: baseline; pneumatic pressure cuff attached to the upper arm	Tests with lightweight and inexpensive miniaturized wireless NIRI device show that the measurement accuracy is comparable to well-established instruments
Atsumori et al., 2009	Pre-frontal cortex while subject performed a word-fluency task	Functional wearable NIRS brain imaging with a prototype during reading	High external validity (but computer task)	1 (adult)	During the task periods, the subject was asked to think of as many words as possible that begin with the Japanese character	Typical changes in oxy-Hb and deoxy-Hb during the task. Therefore, prototype can be used to investigate functions in the prefrontal cortex
Yoshino et al., 2013a	Frontal lobe activations during car acceleration and deceleration	Functional wireless multi-channel system (FOIRE-3000, Shimadzu); 16 sources and 16 detection probes	Very high external validity (field experiment under specific driving conditions)	12 (adults)	Acceleration and deceleration	Results show that vehicle deceleration requires more brain activation, focused in the prefrontal cortex, than does acceleration
Yoshino et al., 2013b	Activation in the frontal lobe during driving operations	Functional wireless multi-channel system (FOIRE-3000, Shimadzu); 16 sources and 16 detection probes	Very high external validity (field experiment under specific driving conditions)	12 (adults)	Resting state, acceleration, deceleration, U-turn, stop	Powerful technique for investigating brain activations outdoors, proving to be sufficiently robust for use in an actual highway driving experiment in the field of intelligent transport systems
Piper et al., 2014	Motor cortex activity during bicycling (left hand gripping)	Functional wireless and mobile NIRS brain imaging during an outdoor activity	Very high external validity (field experiment with specific task conditions)	8 (adults)	Three different exercise conditions: (1) during outdoor bicycle riding; (2) while pedaling on a stationary exercise bicycle; (3) sitting still on a stationary exercise bicycle	Following left hand gripping, a significant decrease in the deoxy-hemoglobin concentration over the contralateral motor cortex could be found for all three conditions; outdoor and indoor conditions showed no significant difference in the shape or amplitude of HbR.
Holper et al., 2014	Simultaneous comparison with EDA; activity of lateral prefrontal cortex during risky decisions	Functional wireless and mobile NIRS brain imaging NIRS machinery with only one light-source	Relatively high external validity (but computer task)	20 (adults)	Risky decision-making task (Christopoulos et al., 2009; Tobler et al., 2009) and baseline recording	Enhanced activation in the lateral prefrontal cortex is related to high-risk decisions; reduced activation in this area is related to low-risk decisions. EDA revealed increasing responses for high-risk decisions

In a later study, Muehlemann et al. (2008) developed a continuous wave near-infrared imaging (NIRI) with four sources and four detectors that was tested in a solid silicone phantom and in an *in-vivo* experiment with one male adult (see Table 2). The results of both phantom and *in-vivo* studies showed that measurement precision with the lightweight and lower-cost miniaturized NIRI is similar to well-established non-mobile NIRS instruments. Testing this prototype on an adult was an important step in the direction of wireless NIRS measurements.

To the best knowledge of the authors, Atsumori et al. (2009) were among the first to carry out a study combining a test for validity and reliability of a new system with an integrated participant task component (see Table 2). The Atsumori team created a small, light and wearable system that covers the participant's forehead in order to measure activation in the prefrontal cortex, and applied it to performing a word fluency tasks. From their study implemented with one Japanese adult, the results showed changes in oxy- and deoxy-hemoglobin that would be typical for this reading task, confirming that the prototype could be used to investigate the prefrontal cortex.

Moreover, a later study applied a wireless, mobile and miniaturized fNIRS prototype (16 channels) for neuroergonomic research (Ayaz et al., 2013). The goal of this prototype was to measure brain activation in naturalistic settings to obtain better knowledge for safety in air traffic control (see Table 2). Though this experiment was executed with only solid and liquid phantoms, the study shows the strong potential for using fNIRS in economic decision-making studies with high external validity.

In 2013, several different studies with wireless prototypes were implemented. One study (*N* = 12 adults) explored frontal lobe activation during car acceleration and deceleration, using a functional wireless multi-channel system (FOIRE-3000, Shimadzu; 16 sources and 16 detectors), and found that vehicle deceleration requires more brain activation—with focus on the prefrontal cortex—than does acceleration (Yoshino et al., 2013a). The study reveals very high external validity by testing participants in a real car in a real-world setting (see Table 2). A second study investigated these first findings regarding brain activations during driving further (Yoshino et al., 2013b). The second study also shows the robustness of using the mobile fNIRS method in a real highway setting. Piper et al. (2014) presented a prototype study of the first wearable multi-channel fNIRS system that could be used for freely moving subjects. In this study, the brain area of interest was the motor cortex activity observed during gripping of the left hand seated at rest, and while cycling outdoors. The experiment was implemented with eight adults and three different exercise conditions (outdoor bicycle riding, riding a stationary exercise bicycle, and sitting still on a bicycle). The results showed a significant decrease in the deoxy-hemoglobin concentration (contralateral motor cortex) for all three cycling conditions, in comparison to the resting conditions. Furthermore, activation in the outdoor condition was not significantly different from riding a stationary exercise bicycle. Therefore, their prototype was assumed to be robust enough for implementation in real-world settings. At this stage, the technology allowed participants in the fNIRS studies to move freely, which is an important precondition for field experiments in naturalistic settings.

#### Mobile NIRS studies with emphasis on economic decision making

Very recently, Holper et al. (2014) presented the first study using wireless and mobile fNIRS machinery with a research question relevant to economics (see Table 2). The researchers tested the activity of the lateral prefrontal cortex during risky decision making, using a simultaneous comparison of the mobile fNIRS system and an EDA device (*N* = 20) in a computer experiment. Results showed that boosted activation in the lateral prefrontal cortex is related to high-risk decisions, and reduced activation in this area with low-risk decisions. Furthermore, the EDA revealed increased response for high-risk decisions. As the first economic decision-making study using mobile fNIRS, the study revealed a number of limitations, most notably that the NIRS machinery had only one light source, and the fictive task was implemented in front of a computer without integrating further situational factors.

However, these prototype studies generally show interesting new tendencies, providing foundation for application of the new wireless and mobile fNIRS techniques as potential measurement methodologies for neuroeconomic studies with high external validity.

Overall, the outline of published studies with stationary and mobile fNIRS machinery presented here indicates that interesting and notable findings exist. However, the area of neuroeconomics is still far away from a systematic integration of fNIRS. This may be due to the lack of a clear indication on the suitability of using fNIRS to study economic decision making. In addition, it may be that neuroeconomists are not trained in application of fNIRS. Regarding these two aspects, we will present a more detailed discussion about the positive and negative aspects of such uses for fNIRS, and develop a decision table to aid in decisions about the suitability of fNIRS in neuroeconomics. Finally, we present a design for a first concept of potential field experiment set-up for neuroeconomic research questions.

## Decision on the suitability of fNIRS methodology for studying neuroeconomics

As shown, very few studies have investigated economic decision making with fNIRS measures, nor have used portable mobile fNIRS measures that allow participants to freely move around in a naturalistic setting. Many researchers are still working on basic methodological studies to optimize the methodology and the analyses of the near-infrared light data. However, the characteristics of the measurements suggest that fNIRS has many strengths, and offers significant potential for neuroeconomics, particularly for research with a high external validity. In order to determine the suitability of using fNIRS, we provide a decision-table for judging the potential for integrating fNIRS studies into neuroeconomic research (see Table 3). Based on this decision-table, neuroeconomic researchers can assess the potential of fNIRS studies for answering their specific research questions.

**Table 3:**
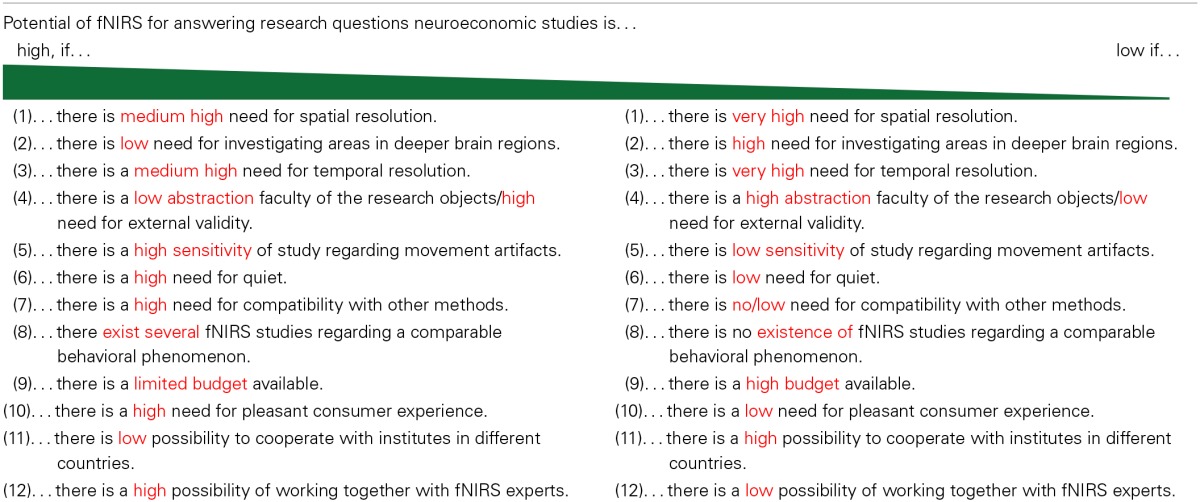
**Potential of fNIRS for neuroeconomics**.

In the following, we elaborate on the various assessment criteria of this decision-table:

1. Spatial resolution: Compared to fMRI, the spatial resolution of fNIRS is less precise. For some research questions, the lower spatial resolution of fNIRS (compared to fMRI) makes it challenging to distinguish cortical areas that are positioned close to each other, so that in earlier multi-channel NIRS studies researchers proposed different algorithms to separate these close regions (Koenraadt et al., 2012; Thanh Hai et al., 2013). These new algorithms are valuable for better identification of cortical sources. Moreover, in contrast to EEG (which measures scalp activity), fNIRS methods are appropriate for specific research questions regarding brain cortical activity (e.g., hypotheses regarding attention/cognition levels and sensory activations). As mentioned in the prior review section, some fNIRS decision studies have investigated cortical and prefrontal processes (e.g., Ernst et al., 2013; Pu et al., 2013; Yoshino et al., 2013a), and can be transferred to economic decision-making questions. Furthermore, some sensory studies (e.g., for advertisement studies) with a focus on perception and imagery processing (e.g., Köchel et al., 2011) are equally transferable.
2. Brain areas in deep brain regions: This aspect is in line with Criteria 1. Cortical and prefrontal areas can be reached effectively by fNIRS studies (e.g., Pu et al., 2012; Ernst et al., 2013; Yoshino et al., 2013a). However, for studies that investigate deeper brain structures (such as the nucleus accumbens) as the concrete region of interest (e.g., Reimann and Bechara, 2010), the fNIRS methodology is less appropriate. This aspect is often mentioned in the limitations of current fNIRS studies (e.g., Pu et al., 2012, 2013) and should be considered when developing a research design for an fNIRS study.
3. Temporal resolution: Generally, in terms of temporal resolution, fNIRS (for the ETG-100 machinery; e.g., 100 ms) is a good compromise between fMRI and EEG/MEG. fNIRS technology supports fast acquisition of data from numerous positions (Ehlis et al., 2005; Huppert et al., 2006; Sitaram et al., 2009). In their NIRS time pressure study, Tsujii and Watanabe (2010) noted activity in the prefrontal cortex (IFC), which demonstrates that fNIRS may provide a good measurement for studies having similar research questions. Tsujii and Watanabe's study (2010) can easily be transferred to neuroeconomic research questions—for example, regarding managers' strategic decision making under time pressure, as well as consumers' decision making under time restrictions. Therefore, this relatively high temporal resolution of fNIRS shows a strong potential for fNIRS methodological investigations regarding time restrictions and limitations in economic settings.
4. Abstraction faculty of the research object: Generally, prior neuroeconomic studies with fMRI investigations have explored economic constructs on a high level of abstraction (e.g., Knutson et al., 2007; Riedl et al., 2010; Kopton et al., 2013). However, for some research questions and concrete economic constructs that need to be examined on a neural basis, limitations exist with regard to the faculty in abstraction. The fNIRS-based driving studies of Yoshino et al. (2013a,b) are a good example of these limitations. fMRI studies could not effectively monitor real driving experiences such as acceleration or deceleration events with external validity. Also, concrete limitations of abstractions exist, with regard to economic constructs—for example, trust in real exchange processes or real-world interpersonal situations. The mobile and wireless capabilities of fNIRS (see also Kober et al., 2013; Yoshino et al., 2013a,b; Piper et al., 2014) indicate optimal possibilities for field experiments in economics.
5. Sensitivity of study regarding movement artifacts: In contrast to fNIRS methodology, fMRI is relatively sensitive to body-motion artifacts (Aihara et al., 2012). The low sensitivity of motion artifacts creates strong potential for fNIRS studies with application in the real world (Nambu et al., 2009; Lloyd-Fox et al., 2010; Kober et al., 2013; Piper et al., 2014). For example, in consumer neuroscience research it is highly relevant to consider consumers' decision-making processes by evaluating not only pictures but also real three-dimensional products (e.g., cars or food items). Studies have often provided evidence that consumers' decision making and affects differ when real products are shown, as compared to pictures (e.g., Bushong et al., 2010). Furthermore, the opportunity to touch or experience real products—in such situations as sitting inside a car or touching clothing—is relevant for some research questions (e.g., Peck and Childers, 2006). These examples suggest that using mobile fNIRS machinery, which is relatively robust to movements, can be a fruitful method for measuring economic decision-making constructs. Nevertheless, researchers who want to investigate economic decision making in real-world settings need to be aware that the mobile prototype machineries are still not immune to movements that are very sudden.
6. Quiet: In contrast to fMRI, fNIRS is an extremely quiet neuroimaging technique (Plichta et al., 2011). This feature gives fNIRS strong relevance for future studies in which auditory sounds play an important role. As an example, a study by Plichta et al. (2011) that shows enhanced activations of sensory brain areas in response to emotional auditory stimuli would be highly transferable to consumer neuroscience studies, as well as to organizational and (neuro-)leadership studies, confirming the significant potential offered by fNIRS studies regarding research questions with auditory stimuli. For instance, it could be both interesting and useful to observe how consumers in real-world shopping situations react to characteristic voices (friendly/unfriendly) of salespeople, or to study the influence of a leader's voice on team or group members.
7. Compatibility with other methods: fNIRS compatibility with other measurements such as EDA is very high (see, for example, Holper et al., 2014). Especially for field experiments with complex stimuli, this possibility could be valuable—for instance, by combining fNIRS with eye-tracking data. Further, because of the quietness of the fNIRS machinery, the method also has very high compatibility with research techniques such as well-established interpersonal market research interviews, which are often used in studies with marketing and strategic management focus. This ability makes the method highly relevant and useful for economic researchers.
8. Existence of several fNIRS studies regarding a comparable behavioral phenomenon (nomological validity): The potential of the fNIRS method for neuroeconomic research studies increases with regard to the number of existing fNIRS studies relative to comparable behavioral phenomenon. Therefore, for basic neuroeconomic research studies using fNIRS methodologies, we suggest a research agenda based on existing inquiries. As mentioned in the review section of the present article, research studies that focus on impulsivity and ADHD syndromes are available, but no studies with a focus on impulsive or compulsive consumer decision making apply fNIRS methodologies. Consequently, exploring consumer impulsivity and compulsivity (e.g., Manolis and Roberts, 2008; Hubert et al., 2013) with the new and still relatively unknown fNIRS measurement has strong potential for generating valuable results for real-world neuroeconomic research. Furthermore, neuroscience studies focused on picture and imagery processing have been implemented, with results suggesting that fNIRS analysis of advertising images could produce valuable new theories for real-world economic applications.
9. Limited budget: In general, fMRI is an expensive neuroimaging method, whereas fNIRS is relatively inexpensive, and the machinery is easily accessible for economic researchers (Shimokawa et al., 2009; Weiskopf, 2012; Kober et al., 2013; Moriguchi and Hiraki, 2013). Therefore, fNIRS studies may have a relatively strong potential for pretest studies, for which researchers would like to test first for expected effects, and have value for economic researchers who have no direct access to fMRI machineries or clinical settings, as well as those who have a relatively low budget. These attributes will enable researchers carrying out fNIRS experiments to include more participants for larger group analyses.
10. Pleasant consumer experience: Marketing or strategic management researchers recognize the value of working with customers of a company, particularly for implementing a study that is pleasant and not uncomfortable for the customers. Doing so prevents conditions that may discourage customers from future interactions with the company, or that may inhibit further buying decisions. This is especially the case for typical market research projects. The use of NIRS is a relatively pleasant experience for participants. As an example, in contrast to EEG measurements, no electrical gel is needed, so that the montage of NIRS optodes is also much faster than the montage of EEG electrodes (Kober et al., 2013). The montage of the machineries for participants with dark hair can take a bit more time, however, because dark hairs between the optodes and the head can cause light attenuation. Though this complication can be diminished by brushing participants' hair out of the way to ensure good skin contact for sources and detectors, the preparation can be time-consuming for researchers (Lloyd-Fox et al., 2010; Holper et al., 2012). For analyses where only the frontal areas are regions of interest, specific fNIRS caps can be used, measuring only the forehead where no hairs disturb the positioning of the optodes (e.g., Atsumori et al., 2009). Overall, NIRS is a very suitable method when there is a need for pleasant consumer experiences.
11. Intercultural study: possibility of cooperating with institutes in different countries: This feature in the decision-list is related to the prior aspect. Building on studies showing that culture has influence on social and interpersonal behavior (Wallendorf and Arnould, 1988; Strombach et al., 2013), it can be anticipated that intercultural aspects have a strong influence on social life and decision making. For real-world conditions where researchers are investigating consumer behaviors in countries other than their own, and where there may be limited ability to cooperate with institutes (China or Africa, for instance), the ability to travel that mobile fNIRS offers to researchers could make it a highly practical tool.
12. Possibility of working with fNIRS experts: For economic researchers who want to answer research questions using fNIRS technology, there is strong advantage in working cooperatively with neuroimaging/optical experts or specific fNIRS experts. In general, the standardization of fNIRS data analysis remains an issue that needs further attention, and many researchers in this area continue to work on their own prototype software to analyze their findings. Therefore, economic researchers who are not yet experts in fNIRS would find particular value in working with experts during the data analysis stage. It should be noted that because there are currently very few fNIRS studies with neuroeconomic focus, comparability between results of different studies continues to be relatively difficult.

In summary, the present discussion, in combination with the elaborated continuum (Table 3), offers neuroeconomic scientists a decision agenda for evaluating the potential of fNIRS methodology, and its usefulness for their specific research questions, aims, and areas of interests.

## Insight into a potential field experiment set-up

As discussed, fNIRS might be a promising and new tool for neuroeconomic research under certain circumstances, especially regarding mobile technologies. In the following, therefore, we will report first insight about a potential experimental set-up using mobile fNIRS in combination with further neurophysiological methods for studies outside the laboratory. The aim of this section is to provide neuroeconomists with greater ability for developing fNIRS studies.

For the application of a mobile fNIRS device in a field experiment, a wearable multi-channel fNIRS system with a specifically developed prefrontal cap is typically used. For our economic decision-making studies (with regard to the measurement restrictions of fNIRS in deeper brain regions), we are mainly interested in the prefrontal areas of the brain.

**Figure 3:**
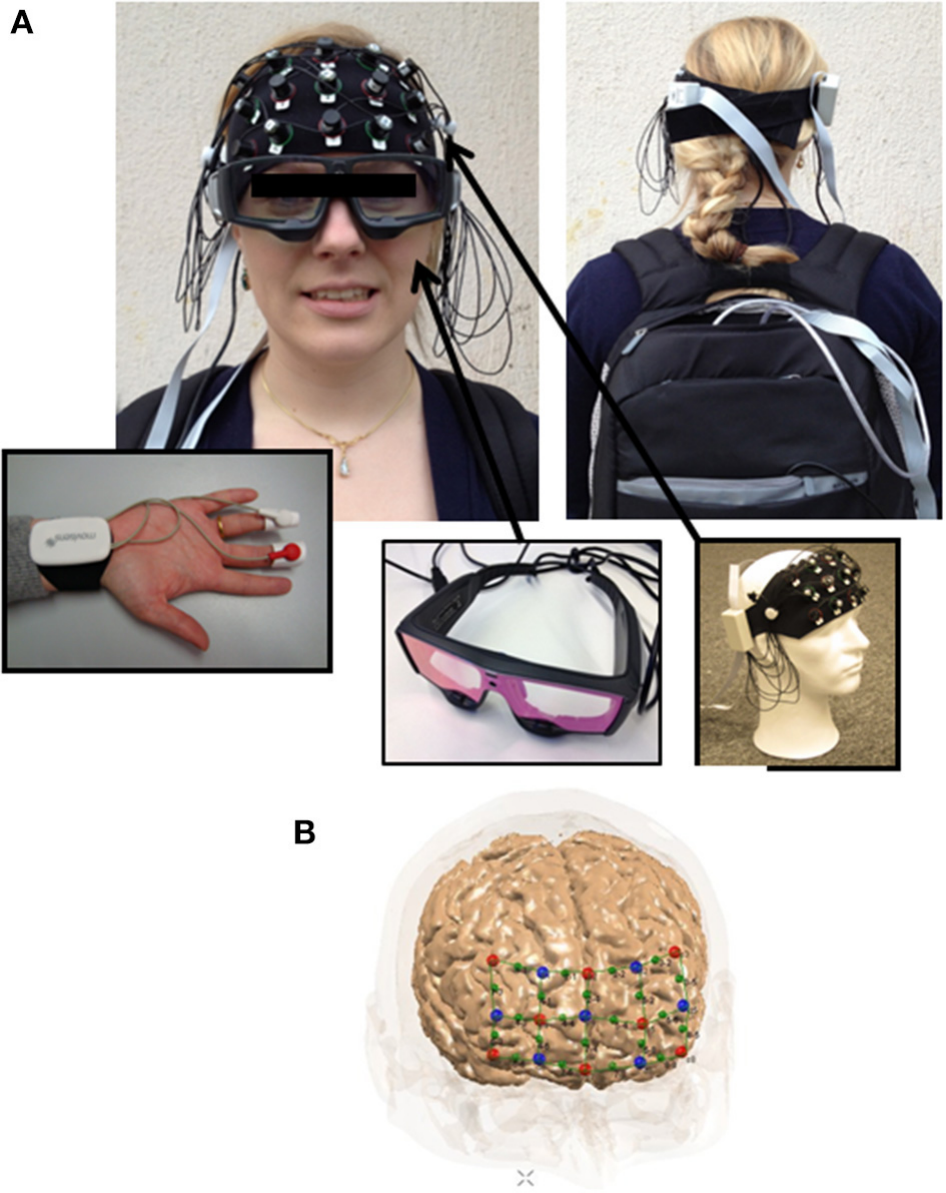
**Potential field experiment set-up. (A)** Exemplary set-up with mobile fNIRS, eye tracking and EDA devide; **(B)** Optode positioning of prefrontal measuring cap specifically developed by NIRx Medizintechnik GmbH, Germany.

Even if scientists keep the design of a field experiment very simple and integrate no complex treatment conditions, questions arise regarding how to control external influencing variables, and how to reconstruct aspects such as consumer behavior for the analysis. These questions are extremely relevant, because consumers in field experiments can move freely and without exact timing conditions, which presents challenges for neurophysiological measurements. For the optimal measurement of consumers' decision making outside the laboratory, a number of reasons can be identified for developing a multifaceted experimental set-up not only with fNIRS measurement, but also with eye-tracking and EDA devices (Figure 3).

The eye-tracking device enables researchers to both follow consumers' eye movements objectively, and to develop a baseline for analysis of the field experiments. Thus, in a field experiment, for example, about consumers' decision making about innovative prototype products such as cars, the eye-tracking data can give important information regarding the stimulus that is being observed by the consumer at a specific time (in seconds) during the decision-making phase. With parallel eye-tracking measurements as baseline and timeline, therefore, participants' individual differences during the experiment outside the laboratory can be controlled. This element of integrating eye-tracking has potential usefulness in neurophysiological field experiments using a portable fNIRS device. Furthermore, comparing fNIRS activation and electrodermal arousal reactions (as additional controls) could be useful for measuring not only eye-tracking but also (“EDA”; e.g., Greenwald et al., 1989) simultaneously with fNIRS (Holper et al., 2014). Prior studies have revealed that specific activations in prefrontal areas have an effect on EDA (Tranel, 2000; Critchley, 2002; Figner and Murphy, 2010; Holper et al., 2014).

Finally, for the successful implementation of experiments outside the laboratory, specific additional operating procedures need to be considered. Consumers should close their eyes (“resting conditions”) before observing objects/stimuli (e.g., car exteriors/interiors), and should walk at a constant speed (to control for potential movement artifacts), in experiments with several rounds and a number of treatment conditions. Moreover, the supervisor of the experiment needs to simultaneously observe each participant, take notes regarding potential outliers (of external variables), and trigger the mobile fNIRS device concerning specific upcoming situations and pre-defined conditions. These operating procedures are relevant for subsequent successful data preprocessing.

## Conclusions

Generally, most prior neuroeconomic studies were implemented with the fMRI scanner, but the fMRI technology also has limitations that have often been criticized—especially concerning the generalizability and the restrictions regarding the integration of situational factors in a lab, and regarding external validity (Braeutigam, 2012). Some of these limiting factors and critical aspects could be countered with new technological tools such as mobile fNIRS devices that support investigation of economic decision making outside the laboratory. Currently, however, few neuroeconomic studies applying mobile NIRS methods are available. The reason for this research gap might be that the use of mobile and wireless NIRS is in its early stages, and many researchers still work on prototypes for optimal data acquisition (e.g., Muehlemann et al., 2008; Atsumori et al., 2009; Piper et al., 2014). The aim of this paper was to demonstrate, and to discuss, the potential that fNIRS methods offer for neuroeconomic research questions in which situational factors outside the laboratory play a major role (e.g., in consumer decision making at the point of sale).

To fulfill our objectives, we reviewed existing studies with relevance to (economic) decision making and presented a decision-table for neuroeconomic researchers that may enable better determination of the suitability of fNIRS for studying neuroeconomics. To the best of our knowledge, this is the first article investigating the fNIRS method as a new and prospective tool for economic research questions outside the laboratory. By integrating studies from various disciplines, we developed a decision-table to support future application of fNIRS methods. Finally, we presented a first concept of a potential field experiment set-up for a neuroeconomic research question.

Overall, this present article shows that further research using (mobile) fNIRS for studies on economic decision making outside the laboratory could be a fruitful avenue. As well, the paper helps to validate the potential of a new method regarding different aspects and to develop a more effective application outside the laboratory.

### Conflict of interest statement

The authors declare that the research was conducted in the absence of any commercial or financial relationships that could be construed as a potential conflict of interest.

## References

[B1] Aihara T. Takeda Y. Takeda K. Yasuda W. Sato T. Otaka Y. (2012). Cortical current source estimation from electroencephalography in combination with near-infrared spectroscopy as a hierarchical prior. Neuroimage 59, 4006–4021 10.1016/j.neuroimage.2011.09.087 22036684

[B2] Ariely D. Berns G. S. (2010). Neuromarketing: the hope and hype of neuroimaging in business. Nat. Rev. Neurosci. 11, 284–292 10.1038/nrn2795 20197790 PMC2875927

[B3] Atsumori H. Kiguchi M. Obata A. Sato H. Katura T. Funane T. (2009). Development of wearable optical topography system for mapping the prefrontal cortex activation. Rev. Sci. Instrum. 80, 043704 10.1063/1.3115207 19405663

[B4] Ayaz H. Onaral B. Izzetoglu K. Shewokis P. A. McKendrick R. Parasuraman R. (2013). Continuous monitoring of brain dynamics with functional near infrared spectroscopy as a tool for neuroergonomic research: empirical examples and a technological development. Front. Hum. Neurosci. 7:871 10.3389/fnhum.2013.00871 24385959 PMC3866520

[B5] Bardhi F. Eckhardt G. M. (2012). Access-based consumption: the case of car sharing. J. Consum. Res. 39, 881–898 10.1086/666376

[B6] Bechara A. Damasio A. R. Damasio H. (2005). The somatic marker hypothesis: a neural theory of economic decision. Games Econ. Behav. 52, 336–372 10.1016/j.geb.2004.06.010

[B7] Belk R. (2010). Sharing. J. Consum. Res. 36, 715–734 10.1086/612649

[B8] Bhatt M. Camerer C. F. (2005). Self-referential thinking and equilibrium as states of mind in games: fMRI evidence. Games Econ. Behav. 52, 424–459 10.1016/j.geb.2005.03.007

[B9] Botsman R. Rogers R. (eds.). (2010). What's Mine Is Yours: The Rise of Collaborative Consumption. New York, NY: HarperCollins

[B10] Boyatzis R. Rochford K. Jack A. (2014). Antagonistic neural networks underlying differentiated leadership roles. Front. Hum. Neurosci. 8:114 10.3389/fnhum.2014.00114 24624074 PMC3941086

[B11] Bozkurt A. Rosen A. Rosen H. Onaral B. (2005). A portable near infrared spectroscopy system for bedside monitoring of newborn brain. Biomed. Eng. Online 4:29 10.1186/1475-925X-4-29 15862131 PMC1112605

[B12] Braeutigam S. (2012). Neural systems supporting and affecting economically relevant behavior. Neurosci. Neuroecon. 1, 11–23 10.2147/NAN.S2503816216681

[B13] Brosch T. Sander D. (2013). Neurocognitive mechanisms underlying value-based decision-making: from core values to economic value. Front. Hum. Neurosci. 7:398 10.3389/fnhum.2013.00398 23898252 PMC3721023

[B14] Bushong B. King L. M. Camerer C. F. Rangel A. (2010). Pavlovian processes in consumer choice: the physical presence of a good increases willingness-to-pay. Am. Econ. Rev. 100, 1–18 10.1257/aer.100.4.1556

[B15] Camerer C. Loewenstein G. Pelec D. (2005). Neuroeconomics: how neuroscience can inform economics. J. Econ. Lit. 43, 9–64 10.1257/0022051053737843

[B16] Christopoulos G. I. Tobler P. N. Bossaerts P. Dolan R. J. Schultz W. (2009). Neural correlates of value, risk, and risk aversion contributing to decision making under risk. J. Neurosci. 29, 12574–12583 10.1523/JNEUROSCI.2614-09.2009 19812332 PMC2794196

[B17] Correia T. Gibson A. Hebden J. (2010). Identification of the optimal wavelengths for optical topography: a photon measurement density function analysis. J. Biomed. Opt. 15, 1–11 10.1117/1.3484747 21054096

[B18] Critchley H. D. (2002). Electrodermal responses: what happens in the brain. Neuroscientist 8, 132–142 10.1177/107385840200800209 11954558

[B19] Davis F. (1989). Perceived usefulness, perceived ease of use, and user acceptance of information technology. Manage. Inform. Syst. Quart. 13, 319–340 10.2307/249008

[B20] Dayan M. Elbanna S. (2011). Antecedents of team intuition and its impact on the success of new product development projects. J. Prod. Innovat. Manag. 28, 159–174 10.1111/j.1540-5885.2011.00868.x

[B21] Deppe M. Schwindt W. Kugel H. Plassmann H. Kenning P. (2005). Nonlinear responses within the medial prefrontal cortex reveal when specific implicit information influences economic decision making. J. Neuroimaging 15, 171–182 10.1177/1051228405275074 15746230

[B22] Dimoka A. (2011). Brain mapping of psychological processes with psychometric scales: an fMRI method for social neuroscience. Neuroimage 54, 263–271 10.1016/j.neuroimage.2010.05.007 20472077

[B23] Duncan A. Meek J. H. Clemence M. Elwell C. Fallon P. Tyszczuk L. (1996). Measurement of cranial optical path length as a function of age using phase resolved near infrared spectroscopy. Pediatr. Res. 39, 889–894 10.1203/00006450-199605000-00025 8726247

[B24] Duncan A. Meek J. H. Clemence M. Elwell C. E. Tyszczuk L. Cope M. (1995). Optical pathlength measurements on adult head, calf and forearm and the head of the newborn infant using phase resolved optical spectroscopy. Phys. Med. Biol. 40, 295–304 10.1088/0031-9155/40/2/007 7708855

[B25] Ehlis A.-C. Herrmann M. J. Wagener A. Fallgatter A. J. (2005). Multi-channel near-infrared spectroscopy detects specific inferior-frontal activation. Biol. Psychol. 69, 315–331 10.1016/j.biopsycho.2004.09.003 15925033

[B26] Ernst L. H. Plichta M. M. Lutz E. Zesewitz A. K. Tupak S. V. Dresler T. (2013). Prefrontal activation patterns of automatic and regulated approach-avoidance reactions—a functional near-infrared spectroscopy (fNIRS) study. Cortex 49, 131–142 10.1016/j.cortex.2011.09.013 22036575

[B27] Fehr E. Fischbacher U. Kosfeld M. (2005). Neuroeconomic foundations of trust and social preferences: initial evidence. Am. Econ. Rev. 95, 346–351 10.1257/000282805774669736 29125275

[B28] Figner B. Murphy R. (2010). Using skin conductance in judgment and decision making research, in A Handbook of Process Tracing Methods for Decision Research, eds Schulte-Mecklenbeck M. Kuehberger A. Ranyard R. (New York, NY: Psychology Press), 163–185

[B29] Funane T. Atsumori H. Katura T. Obata A. N. Sato H. Tanikawa Y. (2013). Quantitative evaluation of deep and shallow tissue layers' contribution to fNIRS signal using multi-distance optodes and independent component analysis. Neuroimage 85, 150–165 10.1016/j.neuroimage.2013.02.026 23439443

[B30] Gassmann O. Enkel E. Chesbrough H. (2010). The future of open innovation as a researchable theory. R&D Manage. J. 40, 213–221 10.1111/j.1467-9310.2010.00605.x

[B31] Gehricke J. G. Polzonetti C. Caburian C. Gratton E. (2013). Prefrontal hemodynamic changes during cigarette smoking in young adult smokers with and without ADHD. Pharmacol. Biochem. Behav. 112, 78–81 10.1016/j.pbb.2013.10.001 24125785 PMC3854671

[B32] Glimcher P. Camerer C. Fehr E. Poldrack R. (2013). Neuroeconomics: Decision Making and the Brain. San Diego, CA: Elsevier

[B33] Greenwald M. K. Cook E. W. Lang P. J. (1989). Affective judgment and psychophysiological response: dimensional covariation in the evaluation of pictorial stimuli. J. Psychophysiol. 3, 51–64

[B34] Hauser J. Tellis G. Griffin A. (2006). Research on innovation: a review and agenda for marketing science. Market. Sci. 25, 687–717 10.1287/mksc.1050.0144

[B35] Hedgcock W. Rao A. R. (2009). Trade-off aversion as an explanation for the attraction effect: a functional magnetic resonance study. J. Market. Res. 46, 1–13 10.1509/jmkr.46.1.1

[B36] Herrmann A. Befurt R. Heitmann M. Berger H. (2007). Alles für die Marke? Produktdesign im Konflikt zwischen einer markenkonformen und einer eigenständigen Produktliniengestaltung. Z. Betrieb. Forsch. 59, 1055–1080

[B38] Holper L. Scholkmann F. Wolf M. (2012). Between-brain connectivity during imitation measured by fNIRS. Neuroimage 63, 212–222 10.1016/j.neuroimage.2012.06.028 22732563

[B37] Holper L. Wolf M. Tobler P. N. (2014). Comparison of functional near-infrared spectroscopy and electrodermal activity in assessing objective versus subjective risk during risky financial decisions. Neuroimage 84, 833–842 10.1016/j.neuroimage.2013.09.047 24096126

[B39] Hubert M. Hubert M. Florack A. Linzmajer M. Kenning P. (2013). Neural correlates of impulsive buying tendencies during perception of product packaging. Psychol. Market. 30, 861–873 10.1002/mar.20651

[B40] Huppert T. J. Hoge R. D. Diamond S. G. Franceschini M. A. Boas D. A. (2006). A temporal comparison of BOLD, ASL and NIRS hemodynamic responses to motor stimuli in adult humans. Neuroimage 29, 368–382 10.1016/j.neuroimage.2005.08.065 16303317 PMC2692693

[B41] Jackson P. A. Kennedy D. O. (2013). The application of near-infrared spectroscopy in nutritional intervention studies. Front. Hum. Neurosci. 7:473 10.3389/fnhum.2013.00473 23964231 PMC3741577

[B42] Jeppesen L. B. Molin M. J. (2003). Consumers as co-developers: learning and innovation outside the firm. Technol. Anal. Strateg. 15, 363–383 10.1080/09537320310001601531

[B43] Jöbsis F. F. (1977). Noninvasive, infrared monitoring of cerebral and myocardial oxygen sufficiency and circulatory parameters. Science 198, 1264–1267 10.1126/science.929199 929199

[B44] Jöbsis-vanderVliet F. F. (1999). Discovery of the near-infrared window into the body and the early development of near-infrared spectroscopy. J. Biomed. Opt. 4, 392–396 10.1117/1.429952 23014610

[B45] Joshi A. W. Sharma S. (2004). Customer knowledge development: antecedents and impact on new product performance. J. Mark. 68, 47–59 10.1509/jmkg.68.4.47.42722

[B46] Kenning P. Kopton I. (2013). Zur Integration neurowissenschaftlicher Erkenntnisse, Theorien und Methoden in die betriebliche Organisationsforschung. Entwicklung einer “Neuroleadership”-Forschungsagenda. Z. Führ. Organ. 6, 388–393

[B47] Kenning P. Linzmajer M. (2011). Consumer neuroscience: an overview of an emerging discipline with implications for consumer policy. J. Consum. Protect. Food Safety 6, 111–125 10.1007/s00003-010-0652-5

[B48] Kenning P. Plassmann H. (2005). Neuroeconomics: an overview of an economic perspective. Brain Res. Bull. 67, 343–354 10.1016/j.brainresbull.2005.07.006 16216680

[B49] Kenning P. Reisch L. (2013). Alternativen zum Informationsparadigma der Verbraucherpolitik: Eine kommentierende Einführung in ein noch dynamisches verbraucherwissenschaftliches Feld mit verbraucherpolitischen Implikationen. J. Verbrauch. Lebensm. 8, 227–230 10.1007/s00003-013-0833-0

[B50] Knutson B. Rick S. Wimmer G. E. Prelec D. Loewenstein G. (2007). Neural predictors of purchase. Neuron 53, 147–156 10.1016/j.neuron.2006.11.010 17196537 PMC1876732

[B51] Kober S. E. Wood G. Kurzmann J. Friedrich E. V. C. Stangl M. Wippel T. (2013). Near-infrared spectroscopy based neurofeedback training increases specific motor imagery related cortical activation compared to sham feedback. Biol. Psychol. 95, 21–30 10.1016/j.biopsycho.2013.05.005 23714227

[B52] Kocsis L. Herrman P. Eke A. (2006). The modified Beer-Lambert law revisited. Phys. Med. Biol. 51, N91–N98 10.1088/0031-9155/51/5/N02 16481677

[B53] Koenraadt K. L. Duysens J. Smeenk M. Keijsers N. L. (2012). Multi-channel NIRS of the primary motor cortex to discriminate hand from food activity. J. Neural Eng. 9:046010 10.1088/1741-2560/9/4/046010 22763344

[B54] Kopton I. Sommer J. Winkelmann A. Riedl R. Kenning P. (2013). Users‘trust building processes during their initial connecting behavior in social networks: behavioral and neural evidence. Proc. Int. Conf. Inform. Syst. 107, 1–12

[B55] Köchel A. Plichta M. M. Schäfer A. Leutgeb V. Scharmüller W. Fallgatter A. (2011). Affective perception and imagery: a NIRS study. Int. J. Psychophysiol. 80, 192–197 10.1016/j.ijpsycho.2011.03.006 21419180

[B56] Krishnan B. C. Dutta S. Jha S. (2013). Effectiveness of exaggerated advertised reference prices: the role of decision time pressure. J. Retail. 89, 105–113 10.1016/j.jretai.2012.11.001

[B57] Kuhnen C. Knutson B. (2005). The neural basis of financial risk taking. Neuron 47, 763–770 10.1016/j.neuron.2005.08.008 16129404

[B58] Lee S. Mysyk A. (2004). The medicalization of compulsive buying. Soc. Sci. Med. 58, 1709–1718 10.1016/S0277-9536(03)00340-X 14990372

[B59] Lloyd-Fox S. Blasi A. Elwell C. E. (2010). Illuminating the developing brain: the past, present and future of functional near infrared spectroscopy. Neurosci. Biobehav. Rev. 34, 269–284 10.1016/j.neubiorev.2009.07.008 19632270

[B60] Luu S. Chau T. (2009). Decoding subjective preference from single-trial near-infrared spectroscopy signals. J. Neural Eng. 6, 1–8 10.1088/1741-2560/6/1/016003 19104138

[B61] Manolis C. Roberts J. A. (2008). Compulsive buying: does it matter how it's measured? J. Econ. Psychol. 29, 555–576 10.1016/j.joep.2007.10.005

[B62] McClure S. M. Laibson D. I. Loewenstein G. Cohen J. D. (2004). Separate neural systems value immediate and delayed monetary rewards. Science 306, 503–507 10.1126/science.1100907 15486304

[B63] Moriguchi Y. Hiraki K. (2013). Prefrontal cortex and executive function in young children: a review of NIRS studies. Front. Hum. Neurosci. 7:867 10.3389/fnhum.2013.00867 24381551 PMC3865781

[B64] Moser S. J. Cutini S. Weber P. Schroeter M. (2009). Right prefrontal brain activation due to Stroop interference is altered in attention-deficit hyperactivity disorder—a functional near-infrared spectroscopy study. Psychiatry Res. 173, 190–195 10.1016/j.pscychresns.2008.10.003 19664910

[B65] Muehlemann T. Haensse D. Wolf M. (2008). Wireless miniaturized *in-vivo* near infrared imaging. Opt. Express 16, 10323–10330 10.1364/OE.16.010323 18607442

[B66] Nambu I. Osu R. Sato M.-A. Ando S. Kawato M. Naito E. (2009). Single-trial reconstruction of finger-pinch forces from human motor-cortical activation measured by near-infrared spectroscopy (NIRS). Neuroimage 47, 628–637 10.1016/j.neuroimage.2009.04.050 19393320

[B67] Nossal R. Kiefer J. Weiss G. H. Bonner R. Taitelbaum H. Havlin S. (1988). Photon migration in layered media. Appl. Opt. 27, 3382–3391 10.1364/AO.27.003382 20539387

[B68] Okada E. Delpy D. T. (2003). Near-infrared light propagation in an adult head model. II. Effect of superficial tissue thickness on the sensitivity of the near-infrared spectroscopy signal. Appl. Opt. 42, 2915–2922 10.1364/AO.42.002915 12790440

[B69] Otero-Lopez J. M. Pol E. V. (2013). Compulsive buying and the five factor model of personality: a facet analysis. Pers. Individ. Dif. 55, 585–590 10.1016/j.paid.2013.05.00510689648

[B70] Peck J. Childers T. L. (2006). If I touch it I have to have it: individual and environmental influences on impulsive purchasing. J. Bus. Res. 59, 765–769 10.1016/j.jbusres.2006.01.014

[B71] Piper S. K. Krueger A. Koch S. P. Mehnert J. Habermehl C. Steinbrink J. (2014). A wearable multi-channel fNIRS system for brain imaging in freely moving subjects. Neuroimage 85, 64–71 10.1016/j.neuroimage.2013.06.062 23810973 PMC3859838

[B72] Plichta M. M. Gerdes A. B. M. Alpers G. W. Harnisch W. Brill S. Wieser M. J. (2011). Auditory cortex activation is modulated by emotion: a functional near-infrared spectroscopy (fNIRS) study. Neuroimage 55, 1200–1207 10.1016/j.neuroimage.2011.01.011 21236348

[B73] Pu S. Nakagome K. Yamada T. Yokoyama K. Matsumura H. Terachi S. (2013). Relationship between prefrontal function during a cognitive task and social functioning in male Japanese workers: a multi-channel near-infrared spectroscopy study. Psychiatry Res. 214, 73–79 10.1016/j.pscychresns.2013.05.011 23932226

[B74] Pu S. Yamada T. Yokoyama K. Matsumura H. Mitani H. Adachi A. (2012). Reduced prefrontal cortex activation during the working memory task associated with poor social functioning in late-onset depression: multi-channel near-infrared spectroscopy study. Psychiatry Res. 203, 222–228 10.1016/j.pscychresns.2012.01.007 22964135

[B75] Reimann M. Bechara A. (2010). The somatic marker framework as a neurological theory of decision-making: review, conceptual comparisons, and future neuroeconomics research. J. Econ. Psychol. 31, 767–776 10.1016/j.joep.2010.03.002

[B76] Reimann M. Castano R. Zaichkowsky J. Bechara A. (2012). Novel versus familiar brands: an analysis of neurophysiology, response latency, and choice. Mark. Lett. 23, 745–759 10.1007/s11002-012-9176-3

[B77] Riedl R. Hubert M. Kenning P. (2010). Are there neural gender differences in online trust? An fMRI study on the perceived trustworthiness of eBay offers. Manage. Inform. Syst. Quar. 34, 397–428 Available online at: http://dl.acm.org/citation.cfm?id=2017458.2017469

[B78] Riedl R. Mohr P. Kenning P. Davis F. Heekeren H. (2014). Trusting humans and avatars: a brain imaging study based on evolution theory. J. Manage. Inform. Syst. 30, 83–113 10.2753/MIS0742-1222300404

[B79] Ruff C. C. Ugazio G. Fehr E. (2013). Changing social norm compliance with noninvasive brain stimulation. Science 342, 482–484 10.1126/science.1241399 24091703

[B80] Sandler-Smith E. Shely E. (2004). The intuitive executive: understanding and applying ‘gut feeling’ in decision-making. Acad. Manag. Exec. 18, 76–91 10.5465/AME.2004.15268692

[B81] Sanfey A. G. Rilling J. K. Aronson J. A. Nystrom L. E. Cohen J. D. (2003). The neural basis of economic decision-making in the ultimatum game. Science 300, 1755–1758 10.1126/science.1082976 12805551

[B82] Sato H. Kiguchi M. Kawaguchi F. Maki A. (2004). Practicality of wavelength selection to improve signal-to-noise ratio in near-infrared spectroscopy. Neuroimage 21, 1554–1562 10.1016/j.neuroimage.2003.12.017 15050579

[B83] Schäfer M. Berens H. Heinze H.-J. Rotte M. (2006). Neural correlates of culturally familiar brands of car manufacturers. Neuroimage 31, 861–865 10.1016/j.neuroimage.2005.12.047 16487728

[B84] Schelkanova I. Toronov V. (2012). Independent component analysis of broadband near-infrared spectroscopy data acquired on adult human head. Biomed. Opt. Express 3, 64–74 10.1364/BOE.3.000064 22254169 PMC3255343

[B85] Schneider S. Christensen A. Häußinger F. B. Fallgatter A. J. Giese M. A. Ehlis A.-C. (2013). Show me how you walk and I tell you how you feel—a functional near-infrared spectroscopy study on emotion perception based on human gait. Neuroimage 85, 380–390 10.1016/j.neuroimage.2013.07.078 23921096

[B86] Scholkmann F. Kleiser S. Metz A. J. Zimmermann R. Pavia J. M. Wolf U. (2013). A review on continuous wave functional near-infrared spectroscopy and imaging instrumentation and methodology. Neuroimage 85, 6–27 10.1016/j.neuroimage.2013.05.004 23684868

[B87] Seligman R. Kirmayer L. J. (2008). Dissociative experience and cultural neuroscience: narrative, metaphor and mechanism. Cult. Med. Psychiatry 32, 31–64 10.1007/s11013-007-9077-8 18213511 PMC5156567

[B88] Shimokawa T. Kinoshita K. Miyagawa K. Misawa T. (2012). A brain information-aided intelligent investment system. Decis. Support Syst. 54, 336–344 10.1016/j.dss.2012.05.04118958272

[B89] Shimokawa T. Suzuki K. Misawa T. Miyagawa K. (2009). Predictability of investment behavior from brain information measured by functional near-infrared spectroscopy: a Bayesian neural network model. Neuroscience 161, 347–358 10.1016/j.neuroscience.2009.02.079 19303915

[B90] Singer T. Fehr E. (2005). The neuroeconomics of mind reading and empathy. Am. Econ. Rev. 95, 340–345 10.1257/000282805774670103 29125271

[B91] Sitaram R. Caria A. Birbaumer N. (2009). Hemodynamic brain-computer interfaces for communication and rehabilitation. Neural Netw. 22, 1320–1328 10.1016/j.neunet.2009.05.009 19524399

[B92] Stevens G. Burley J. (2003). Piloting the rocket of radical innovation. Res. Technol. Manage. 46, 16–26 10.1109/EMR.2004.25114

[B93] Strombach T. Jin J. Weber B. Kenning P. Shen Q. Ma Q. (2013). Charity begins at home: cultural differences in social discounting and generosity. J. Behav. Decis. Making 27, 235–245 10.1002/bdm.1802

[B94] Thanh Hai N. Cuong N. Q. Dang Khoa T. Q. Toi V. V. (2013). Temporal hemodynamic classification of two hands tapping using functional near-infrared spectroscopy. Front. Hum. Neurosci. 7:516 10.3389/fnhum.2013.00516 24032008 PMC3759001

[B94a] Tobler P. N. Christopoulos G. I. O'Doherty J. P. Dolan R. J. Schultz W. (2009). Risk-dependent reward value signal in human prefrontal cortex. Proc. Natl. Acad. Sci. 106, 7185–7190 10.1073/pnas.0809599106 19369207 PMC2678436

[B95] Tranel D. (2000). Electrodermal activity in cognitive neuroscience: neuroanatomical and neuropsychological correlate, in Cognitive Neuroscience in Emotion, eds Lane R. Nadel L. (New York, NY: Oxford University Press), 192–224

[B96] Tsujii T. Watanabe S. (2010). Neural correlates of belief-bias reasoning under time pressure: a near-infrared spectroscopy study. Neuroimage 50, 1320–1326 10.1016/j.neuroimage.2010.01.026 20080190

[B97] Villringer A. Planck J. Hock C. Schleinkofer L. Dirnagl U. (1993). Near infrared spectroscopy (NIRS): a new tool to study hemodynamic canges during activation of brain function in human adults. Neurosci. Lett. 154, 101–104 10.1016/0304-3940(93)90181-J 8361619

[B98] von Hippel E. Katz R. (2002). Shifting innovation to users via toolkits. Manage. Sci. 48, 821–833 10.1287/mnsc.48.7.821.2817

[B99] Wallendorf M. Arnould E. J. (1988). My favorite things: a cross-cultural inquiry into object attachment, possessiveness, and social linkage. J. Consum. Res. 14, 531–547 10.1086/209134

[B100] Weiskopf N. (2012). Real-time fMRI and its application to neurofeedback. Neuroimage 62, 682–692 10.1016/j.neuroimage.2011.10.009 22019880

[B101] Yamashita Y. Maki A. Koizumi H. (2001). Wavelength dependence of the precision of noninvasive optical measurement of oxy-, deoxy-, and total-hemoglobin concentration. Med. Phys. 28, 1108–1114 10.1118/1.1373401 11439480

[B102] Yoon C. Gutchess A. H. Feinberg F. Polk T. A. (2006). A functional magnetic resonance imaging study of neural dissociations between brand and person judgments. J. Consum. Res. 33, 31–40 10.1086/504132

[B103] Yoshino K. Oka N. Yamamoto K. Takahashi H. Kato T. (2013a). Correlation of prefrontal cortical activation with changing speeds in actual driving, a vector-based functional near-infrared spectroscopy study. Front. Hum. Neurosci. 7:895 10.3389/fnhum.2013.00895 24399953 PMC3872330

[B104] Yoshino K. Oka N. Yamamoto K. Takahashi H. Kato T. (2013b). Functional brain imaging using near-infrared spectroscopy during actual driving on an expressway. Front. Hum. Neurosci. 7:882 10.3389/fnhum.2013.00882 24399949 PMC3871711

